# Supplementary material for: Impact of the Substitution Pattern at the Basic Center and Geometry of the Amine Fragment on 5-HT6 and D3R Affinity in the 1H-Pyrrolo[3,2-c]quinoline Series
Source: Molecules. 2023 Jan 21;28(3):1096. doi: 10.3390/molecules28031096 (PMC9920808; doi:10.3390/molecules28031096)
Supplement: Supplementary file 1 [file molecules-28-01096-s001.zip › molecules-2140092-supplementary.pdf]

Impact of the substitution pattern at the basic center and geometry of the amine fragment on the 5-HT_6 and D_3Rs affinity in the 1*H*-pyrrolo[3,2-*c*]quinoline series

Katarzyna Grychowska,[1,] * Wojciech Pietruś,[2] Ludmiła Kulawik,[1] Ophélie Bento,[3] Grzegorz Satała,[2] Xavier Bantreil,[4,5] Frédéric Lamaty,[4] Andrzej J. Bojarski,[2] Joanna Gołębiowska,[2] Agnieszka Nikiforuk,[2] Philippe Marin,[3] Séverine Chaumont-Dubel,[3] Rafał Kurczab[2] and Paweł Zajdel[1]

[1] *Faculty of Pharmacy Jagiellonian University Medical College, 9 Medyczna Str., 30-688 Kraków, Poland*

[2] *Maj Institute of Pharmacology, Polish Academy of Sciences, 12 Smętna Str., 31-324 Kraków, Poland*

[3] *IGF, Université de Montpellier, CNRS INSERM, 34094 Montpellier, France*

[4] *IBMM, Université de Montpellier, CNRS, ENSCM, Montpellier, France*

[5] *Institut Universitaire de France (IUF), Paris, France*

*Corresponding authors

e-mail: katarzyna.grychowska@uj.edu.pl

Table of contents

Figure S1. Superposition of the binding modes of compounds **9**, **12** and **13**............................. 3

^{1}H NMR and ^{13}C NMR spectra of representative compounds.. 4

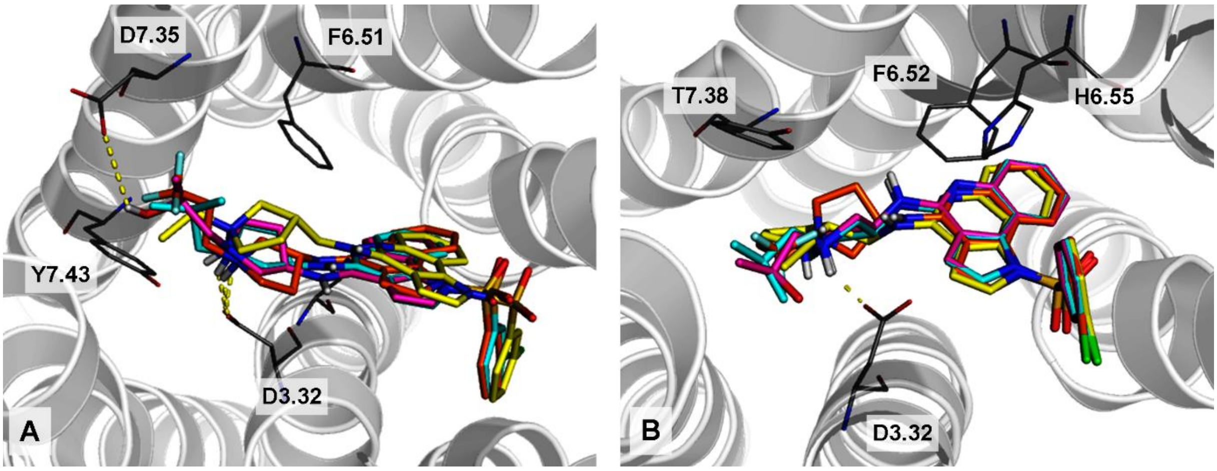

Figure S1. Superposition of the binding modes of **9** (magenta)**, 12** (orange) and **PZ-1643** (cyan) in the 5-HT_6 (A) and D_3R binding sites.

^{1}H NMR and ^{13}C NMR spectra of representative compounds

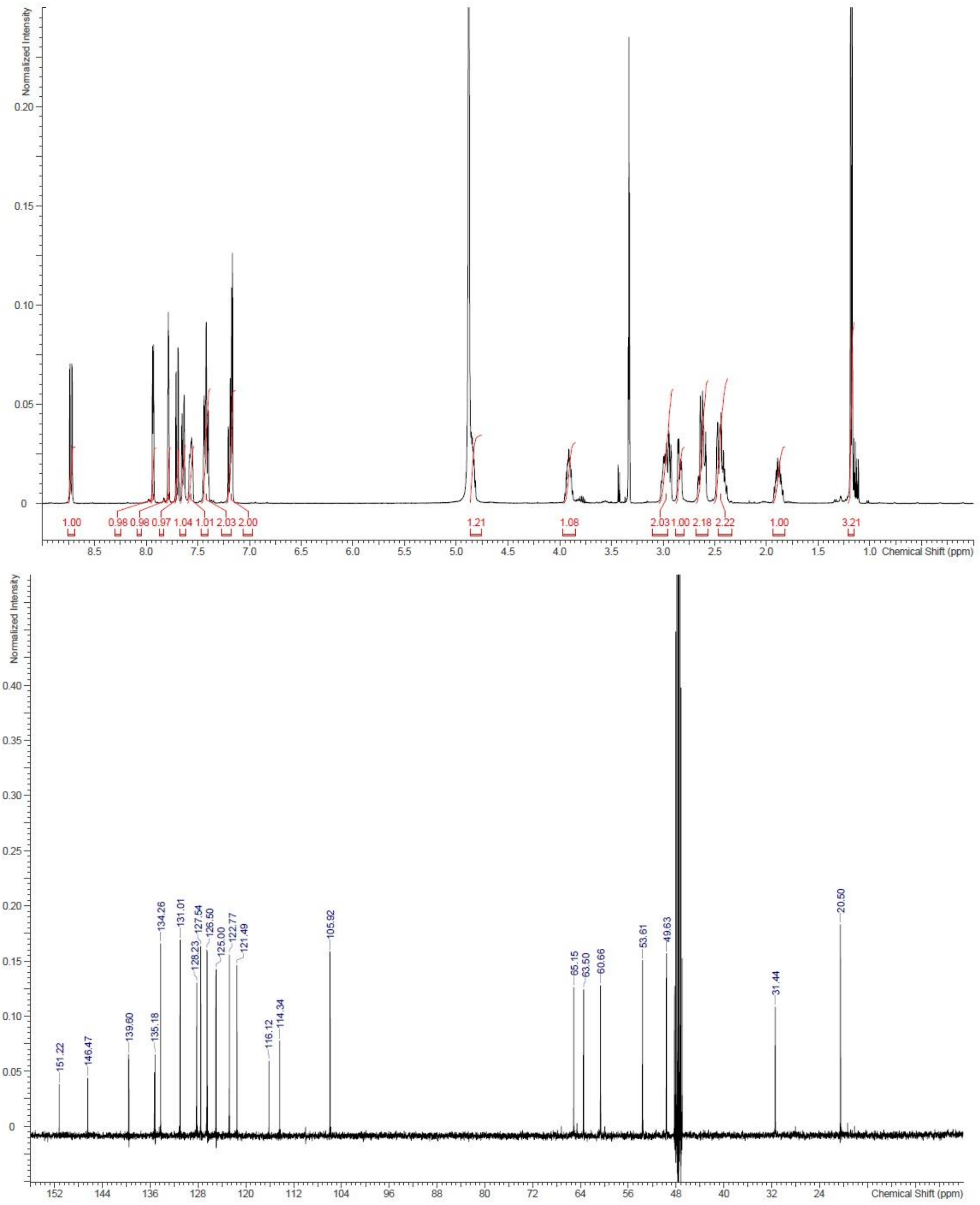

Figure S2. S)-1-((S)-3-((1-((3-chlorophenyl)sulfonyl)-1H-pyrrolo[3,2-c]quinolin-4-yl)amino)pyrrolidin-1-yl)propan-2-ol **8.**

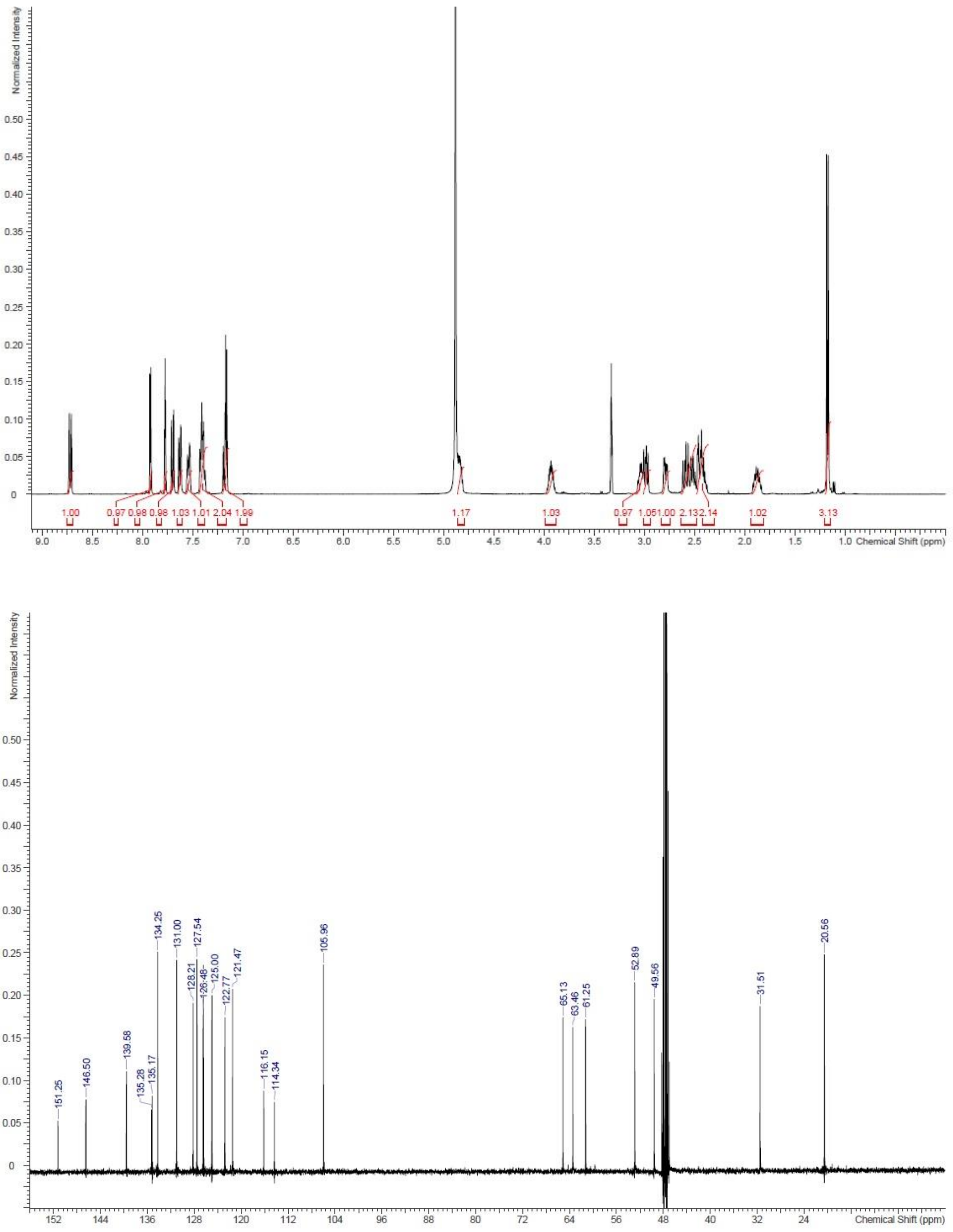

Figure S3. (R)-1-((S)-3-((1-((3-chlorophenyl)sulfonyl)-1H-pyrrolo[3,2-c]quinolin-4-yl)amino)pyrrolidin-1-yl)propan-2-ol **9**

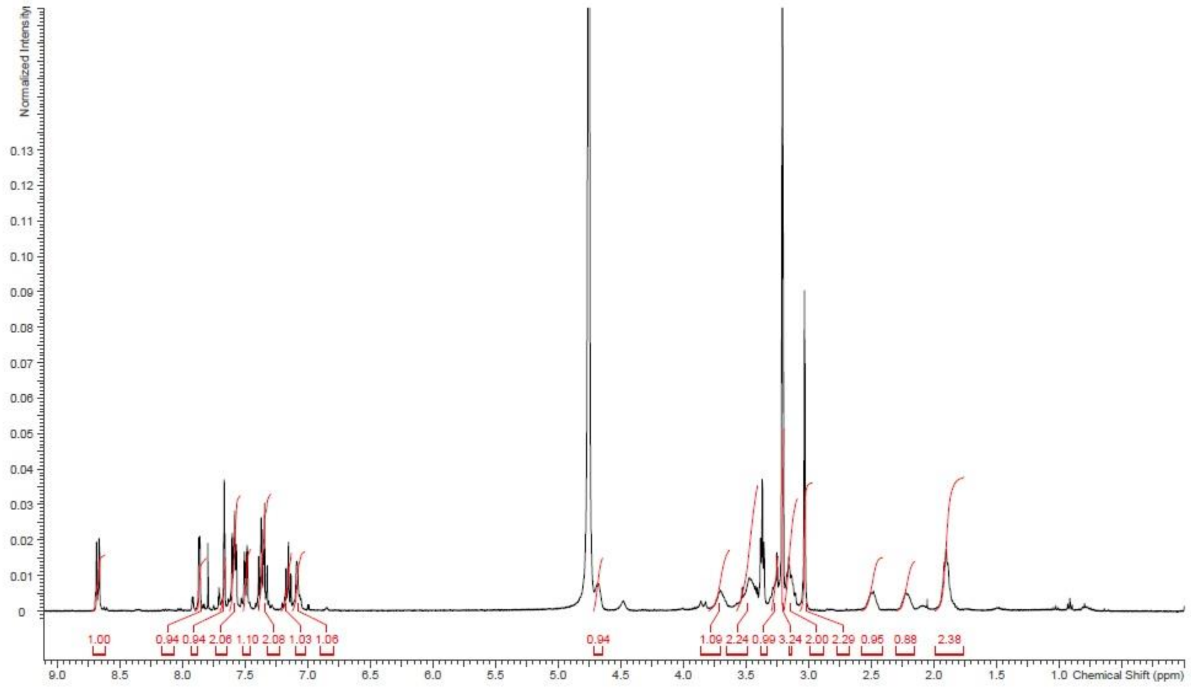

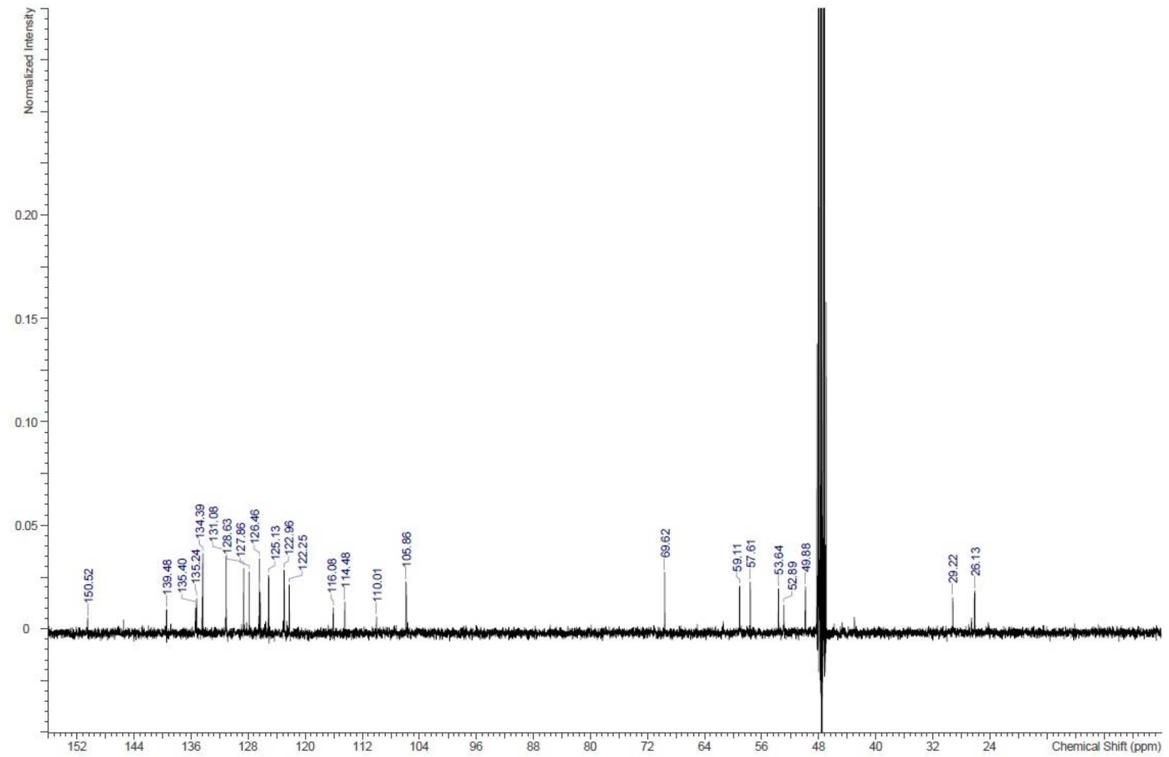

Figure S4. (S)-1-((3-chlorophenyl)sulfonyl)-N-(1-(3-methoxypropyl)pyrrolidin-3-yl)-1H-pyrrolo[3,2-c]quinolin-4-amine **11**

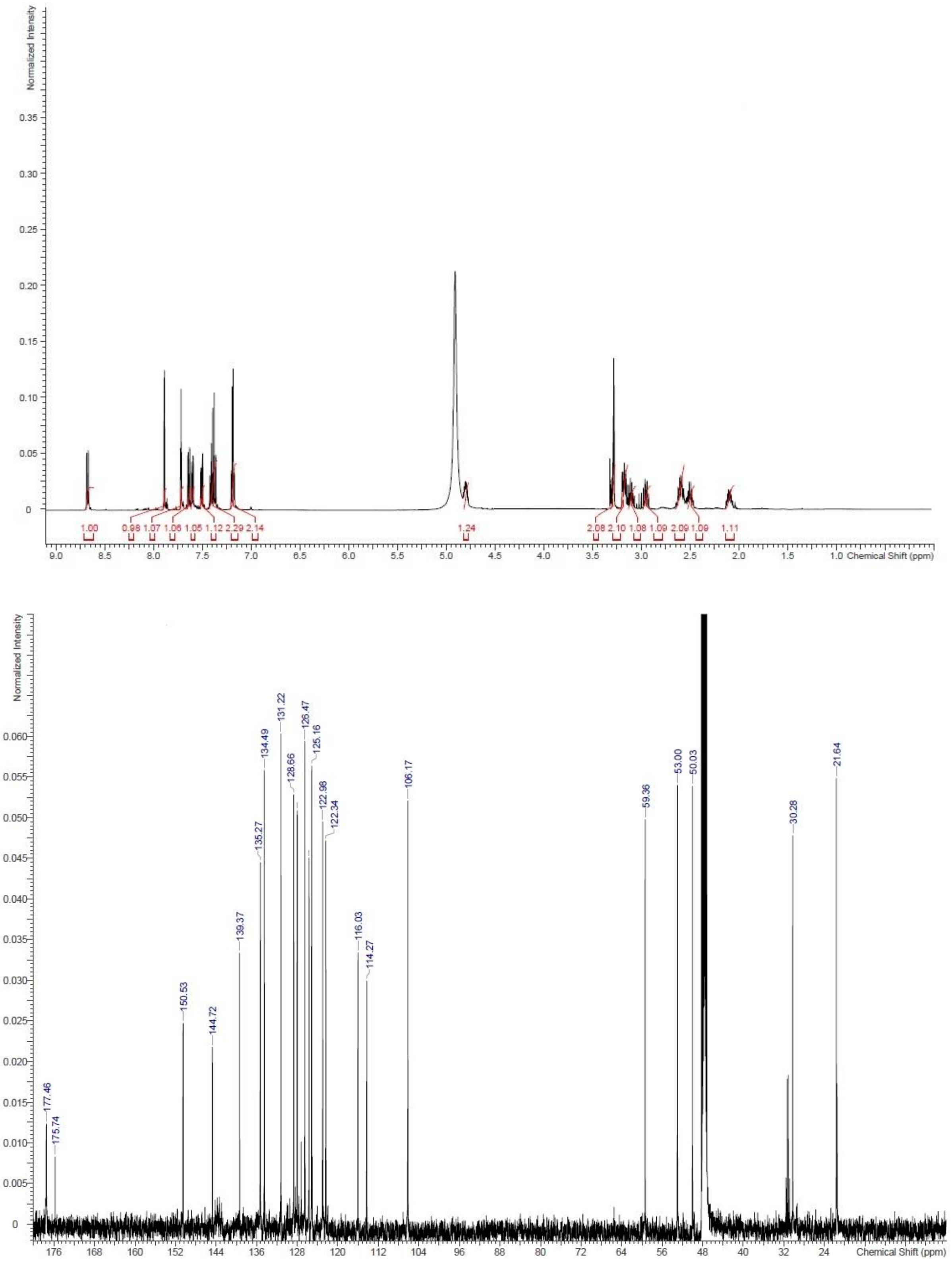

Figure S5. (S)-1-((3-chlorophenyl)sulfonyl)-N-(1-(3,3,3-trifluoropropyl)pyrrolidin-3-yl)-1H-pyrrolo[3,2-c]quinolin-4-amine **12**

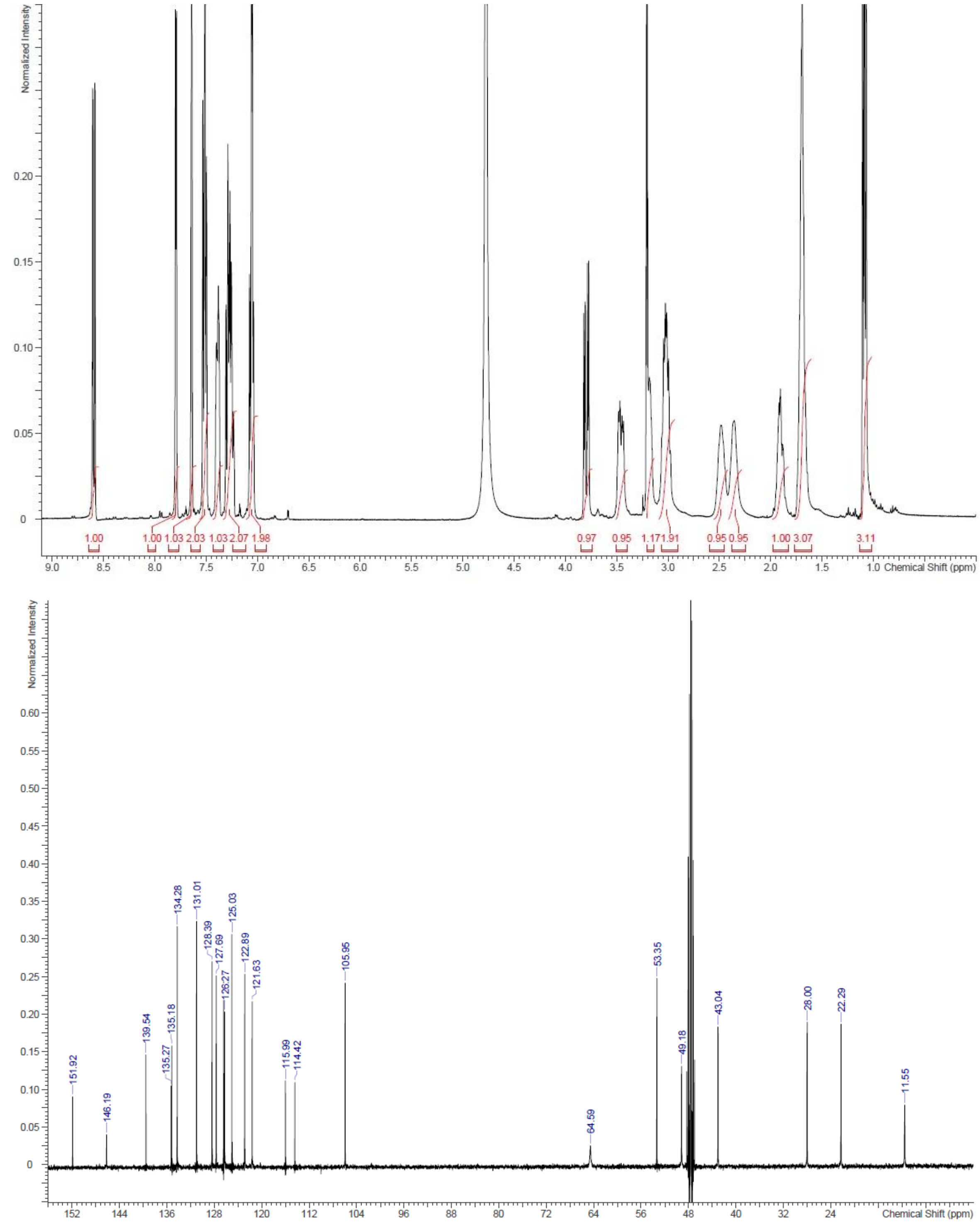

Figure S6. (R)-1-((3-chlorophenyl)sulfonyl)-N-((1-ethylpyrrolidin-2-yl)methyl)-1H-pyrrolo[3,2-c]quinolin-4-amine hydrochloride **13**

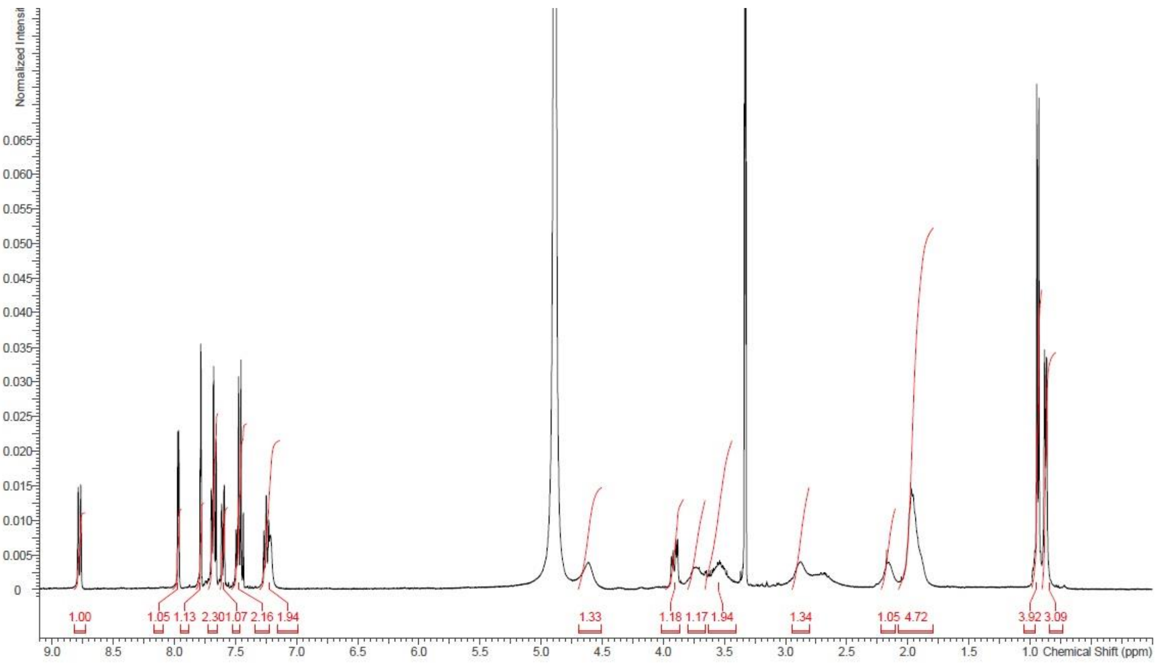

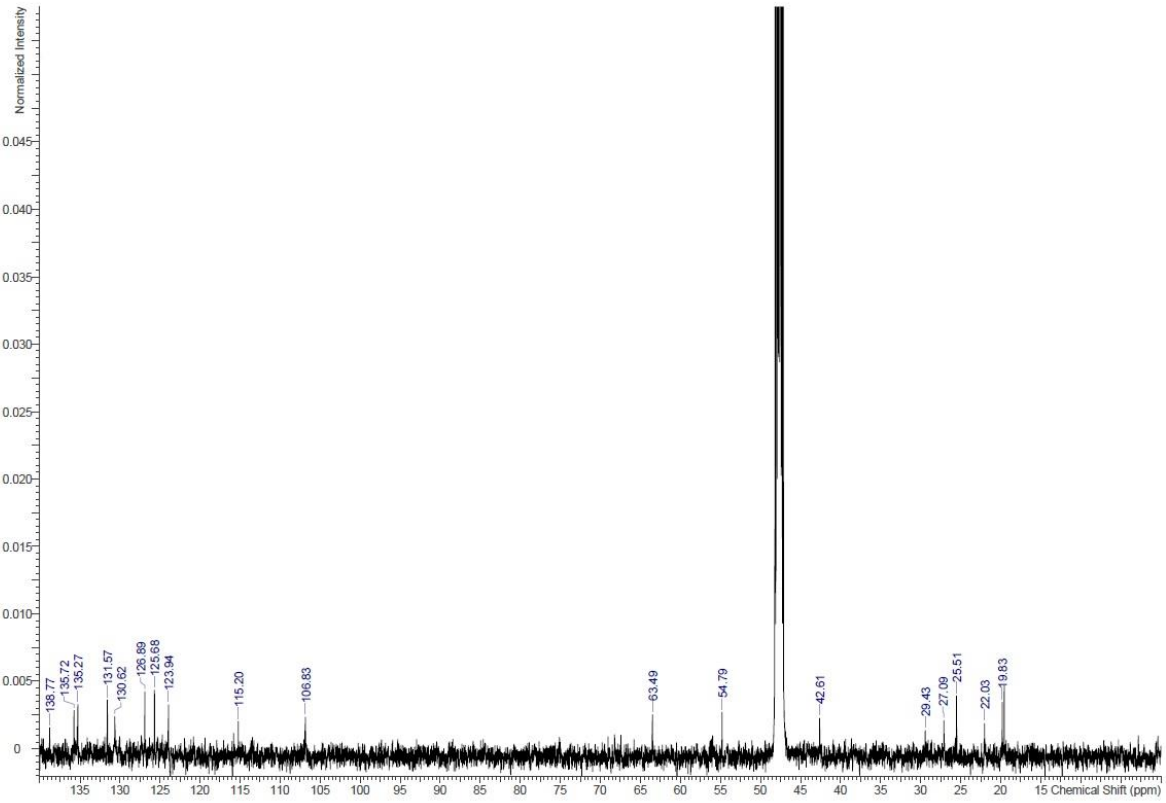

Figure S7. (R)-1-((3-chlorophenyl)sulfonyl)-N-((1-isobutylpyrrolidin-2-yl)methyl)-1H-pyrrolo[3,2-c]quinolin-4-amine hydrochloride **17**
